# *SOX9* Duplication Linked to Intersex in Deer

**DOI:** 10.1371/journal.pone.0073734

**Published:** 2013-09-06

**Authors:** Regina Kropatsch, Gabriele Dekomien, Denis A. Akkad, Wanda M. Gerding, Elisabeth Petrasch-Parwez, Neil D. Young, Janine Altmüller, Peter Nürnberg, Robin B. Gasser, Jörg T. Epplen

**Affiliations:** 1 Human Genetics, Ruhr University, Bochum, Germany; 2 Neuroanatomy and Molecular Brain Research, Ruhr University, Bochum, Germany; 3 Faculty of Veterinary Science, the University of Melbourne, Parkville, Victoria, Australia; 4 Cologne Center for Genomics, University of Cologne, Cologne, Germany; Baylor College of Medicine, United States of America

## Abstract

A complex network of genes determines sex in mammals. Here, we studied a European roe deer with an intersex phenotype that was consistent with a XY genotype with incomplete male-determination. Whole genome sequencing and quantitative real-time PCR analyses revealed a triple dose of the *SOX9* gene, allowing insights into a new genetic defect in a wild animal.

## Introduction

Sexual development in mammals depends on a complex network of regulatory genes controlled by the presence or absence of the testis-determining gene *SRY* (sex-determining region on the Y chromosome) [1]. The protein encoded by *SRY* up-regulates the expression of transcription factor SOX9. The activation and maintenance of SOX9 expression triggers other genes (*e.g. SOX8*) required for testis and male differentiation, whereas SOX9 itself actively suppresses the expression of genes involved in ovarian development. In the absence of SRY, the alternative, female pathway is activated, resulting in ovary formation [2]. Derangements in these processes cause malformations of internal and external genitalia, varying from sexual ambiguity to complete sex reversal. The sex reversal syndrome in humans and other mammals is a congenital abnormality, characterized by inconsistency between chromosomal and gonadal sex [3]. In humans, sex reversal is a rare disorder of sterile XX males or XY females, with an incidence of one in 20,000-25,000 males [4]. XX sex reversals occur in different mammals, including European roe deer ( *Capreolus* *capreolus* ) [5]. Phenotypes can range from females carrying antlers to true hermaphrodites, although pronounced sexual dimorphism is usually evident [5]. *C* *. capreolus* has a high population density in Europe [6] and is the commonest cloven-hoofed game in Germany [7]; nonetheless, encountering sexual defects in natural populations appears to be extremely rare. Here, we studied a roe deer specimen with an intersex phenotype, resulting from incomplete male-determination. In order to explore the genetic basis of this defect, we sequenced the genome of roe deer.

## Results

**Figure 1:**
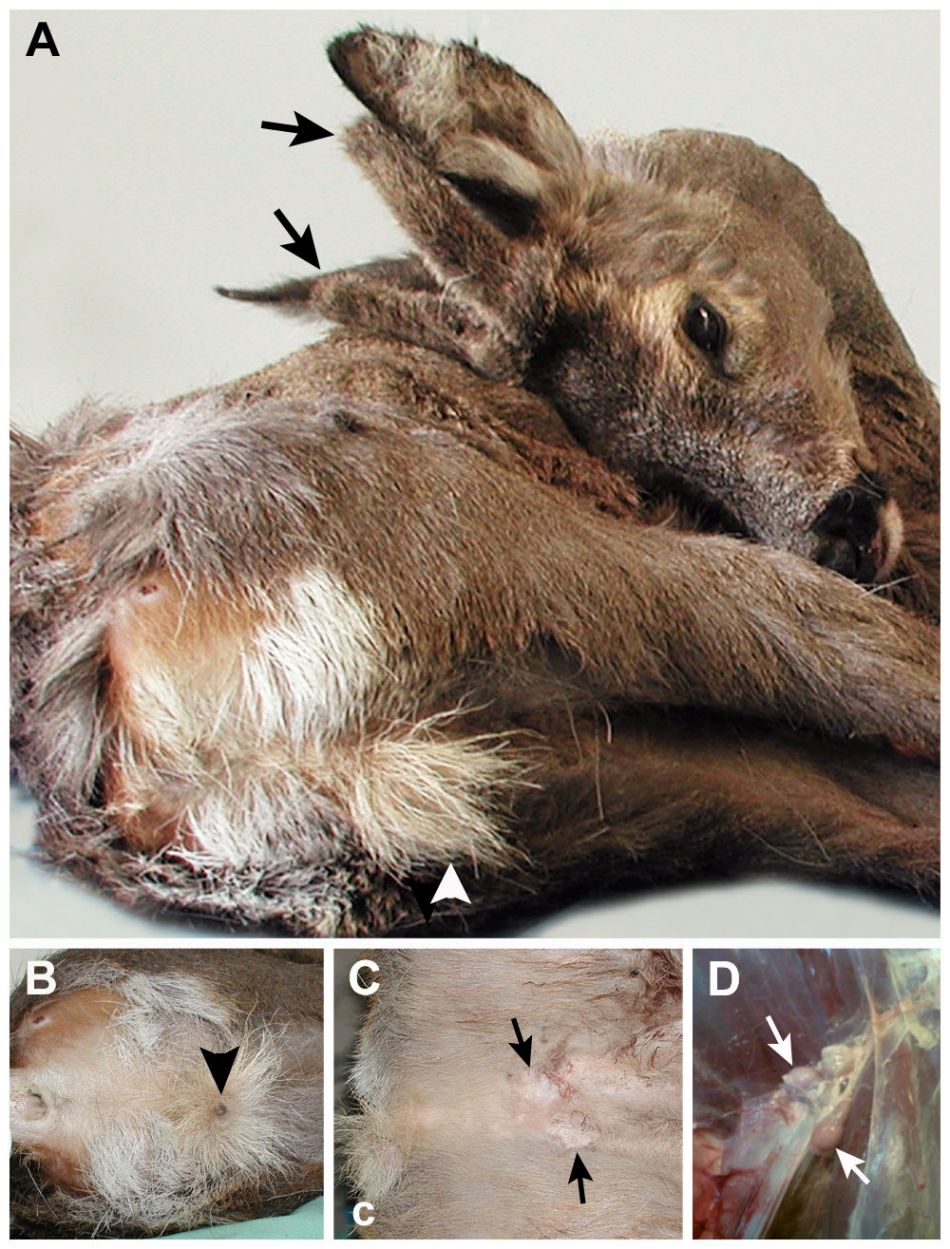
European roe deer ( *C* *. capreolus* ) specimen with intersexual appearance. (**A**) Habitus of the one-year old deer with short unbranched antlers (arrows) and a displaced penis opening in a brush appearing from the distance as a *Schürze* (arrowhead, characteristic tuft of hair in females normally located just above the vaginal entry). (**B**) Closer view of the back of the investigated deer, including anus and retro-posed penis with urethral opening (arrowhead). (**C**) Inguinal testicles under the abdominal skin (arrows). (**D**) *Situs* of the reproductive tract including the left and right small testicles (arrows) which had not descended into a scrotum; the right epididymis is also discernible.

Fortuitously, we had access to a bagged, one year-old *C* *. capreolus* with a distinct intersex phenotype (Figure 1). It had cranial outgrowths typical of a buck and a rear phenotype characteristic of a doe. Close inspection revealed no vaginal opening, abdominal testes and a retro-posed pizzle. In contrast to a healthy male *C* *. capreolus* , there was no evidence of the single copy *amelogenin* (*AMEL*) gene [8] in the Y chromosome-specific region, indicating a female sex chromosome status, in spite of male external genitalia.

We then proceeded to explore the genetic basis of this defect by investigating ten genes [sex-determining region Y gene (*SRY*), SRY-related HMG-box gene 3 (*SOX3*), SRY-related HMG box gene 9 (*SOX9*), SRY-related HMG box gene 10 (*SOX10*), R-spondin 1 (*RSPO1*), forkhead transcription factor FOXL2 (*FOXL2*), double-sex- and MAB3-related transcription factor 1 (*DMRT1*), fibroblast growth factor 9 (FGF9), Wilms tumor gene 1 (*WT1*), androgen receptor (AR)] which are all known, in humans and mice, to be involved in sex determination or SRY-negative XX sex reversal, if mutated [3]. Since no sequence data are presently available for roe deer, we sequenced genomic DNA from a healthy male European roe deer using Illumina technology to produce 920 million paired-end reads (21-fold coverage). We assembled contigs from these reads, identified the aforementioned ten full-length genes of roe deer by comparison with homologous genes in the bovine genome (90-95% similarity), and independently sequenced each of these genes in the intersex, male and female control deer to verify the sequences in assembled contigs. We identified 42 sequence variations in *SOX3*, *SOX9*, *SOX10*, *RSPO1*, *FOXL2*, *DMRT1*, *FGF9, WT1* and *AR* (Table 1) but none in *SRY*. Most of these alterations (*n*=25) are located intronically or in untranslated regions, and do not involve splice sites or regulatory regions. All variations in exonic regions are silent, except for two, which were also present in healthy roe deer controls, thus excluding them as a causal link to intersexuality in the present roe deer case. Two sequence variations identified in the X-chromosomally-located genes *AR* and *SOX3* were detected in a heterozygous state in the intersex deer. Together with the lack of *AMEL* and *SRY* gene sequences, these data indicate the sole presence of two X chromosomes, representing the female sex chromosome status.

**Table 1:** Sequence variations identified in roe deer genes involved in sex determination.

**Gene**	**Exon**	**Sequence variation**	**Amino acid exchange**	**Individual**		
				**Intersex**	♀ **control**	♂ **control**
***AR***	1	c.1143C>T	p.Tyr381=	TT	TT	
	2	c.1683C>A	p-Ala561=	CC	CA	
	5	c.2100C>T	p.Phe700=	CT	CC	
	6	IVS6+68A>T	-	AA	TT	
***DMRT1***	1	c.93G>C	p.Gly31=	CC	GG	GG
		c.129_130insGGC	p.Gly43_Ser44insGly	WT WT	WT WT	WT insGGC
	2	IVS1-90G>A	-	GG	AA	
		c.501T>C	p.Ala167=	TT	TC	
	4	c.813T>C	p.His271=	CC	TT	
		IVS5+36A>G	-	GG	AG	
***FGF9***	2	c.330A>G	p.Val110=	GG	GG	GG
	3	c.594C>T	p.Pro198=	CC	CC	CT
***FOXL2***	1	c. *1G>A	-	GA	GG	AA
***RSPO1***	2	c. -8G>A	-	GA	GG	GA
	6	IVS5-72C>T	-	CT	TT	
***SOX3***	1	c.600A>G	p.Glu200=	AG	GG	GG
***SOX9***	5UTR	c. -891C>G	-	CG	CG	CG
		c. -841C>T	-	CC	CT	CT
		c. -740G>A	-	AA	GA	GA
		c. -424C>G	-	GG	GG	GG
		c. -411A>C	-	AA	CC	CC
		c. -271G>C	-	CC	GC	GC
	2	IVS1+382_383insC	-	WT WT	C C	WT C
		IVS1+449G>A	-	GG	GA	GA
		IVS1-182G>T	-	TT	TT	
		c.528G>A	p.Pro176=	AA	GA	
	3	c.1539G>A	p.Pro513=	GA	GA	GA
***SOX10***	4	c.960T>C	p.Tyr320=	CC	TT	
		c.1011C>T	p.Ser337=	CT	CC	
		c.1252A>G	p.Ser417Gly	AG	AG	
		c. *7G>A	-	GG	GA	
***WT1***	1	c. -14T>G	-	TG	TT	TT
		IVS1+30G>T	-	GT	GG	GG
		IVS1+45C>T	-	CT	CC	CC
	3	IVS2-20G>C	-	GG	CC	GG
	4	IVS5+33C>T	-	CC	TT	TT
	5	IVS5+57G>A	-	GG	GG	GA
	6	c.810A>G	p.Thr270=	AA	AG	AA
		c.849T>C	p.Cys283=	TT	CC	CC
		IVS6+47A>G	-	AA	GG	AG
	8	IVS7-18_-17delAT	-	delAT delAT	WT WT	WT WT
	10	IVS9-157C>A	-	CC	AA	CC

For insertion or deletion sequence variations, the wild type allele is indicated by “WT”. Blank fields account for lacking amplification products. As indicated by (-), no amino acid exchange was identified for the particular sequence variation.

**Figure 2:**
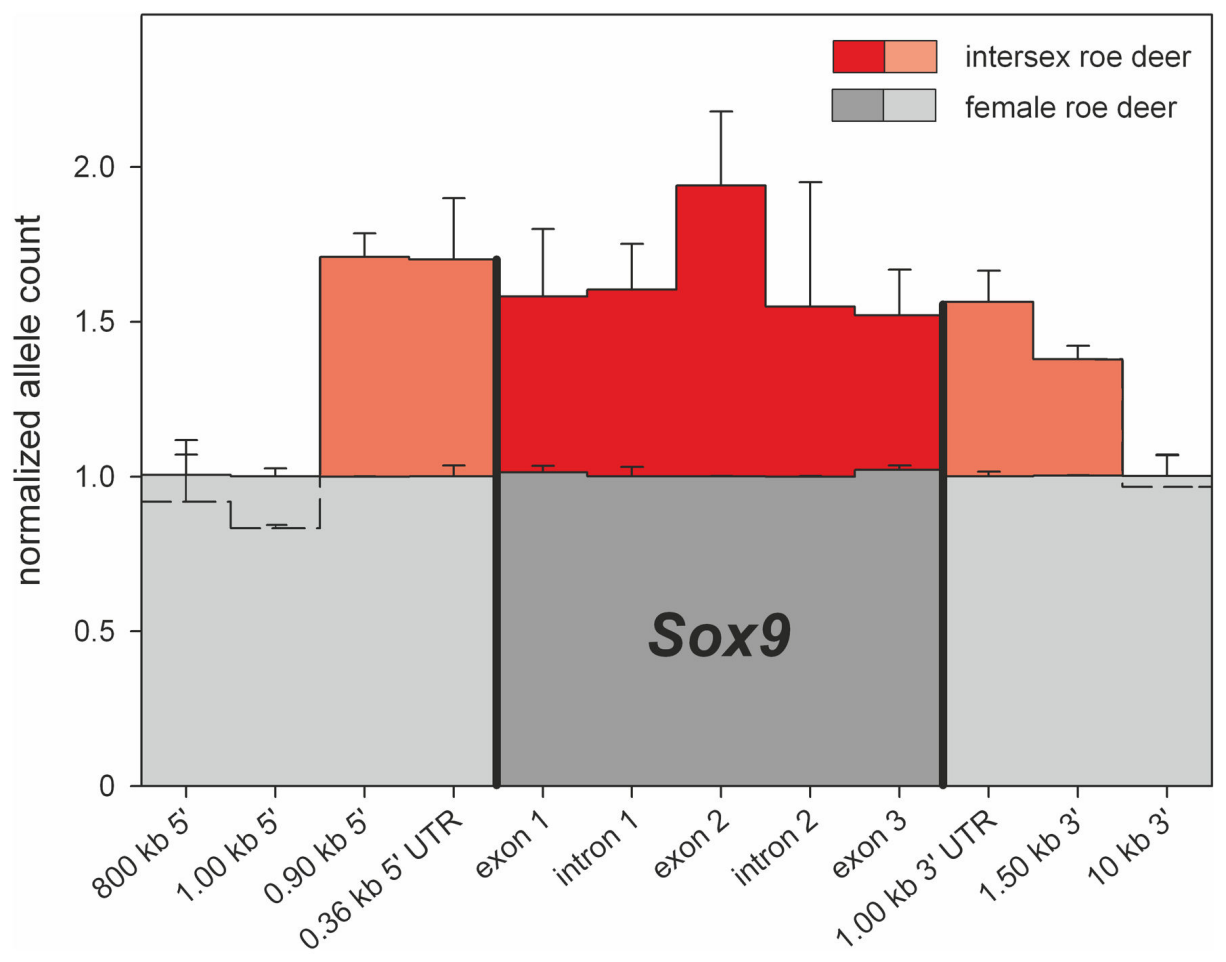
Analysis of copy number variation by quantitative real-time PCR for the *SOX9* gene, including exons, introns, 5’ and 3’ untranslated regions in the intersex roe deer as compared with a healthy female control. In the intersex, the entire *SOX9* gene and regions 5’ and 3’ of the gene is duplicated (red bars) as compared with the female control showing normal gene dosage for all regions investigated (grey bars).

Since in humans, female-to-male sex reversal can be due to a translocation of Y chromosome sequences to an X chromosome encompassing *SRY* [9], we analyzed this gene. Yet, no *SRY*-specific fragment was detectable by PCR in the intersex deer, in contrast to XY male controls. Since the copy number of some genes have been shown to interfere with normal sexual differentiation [10], we investigated the dosages for *SOX3*, *SOX9*, *SOX10, RSPO1, DMRT1*, *WT1, RSPO1, FOXL2* and *AR* using selected PCR amplicons for initial duplication deletion screening via quantitative real-time PCR analysis. While the copy numbers were identical in the intersex as compared with male and female control deer, the *SOX9* gene showed a triple dosage compared with the controls. In order to exclude the possible involvement of a pseudogene, in-depth analyses of all three individual exons, both introns and the 5’- and 3’-untranslated regions were performed for the intersex as well as for one female and one male control deer. The analysis revealed the presence of three copies of the *SOX9* gene in the intersex roe deer, in contrast to controls. Also the dosage of the 5’- and 3’-untranslated regions of *SOX9* was increased by 3-fold, but further 5’-upstream and 3’-downstream regions showed normal, double dosage in the intersex individual (Figure 2). Duplication breakpoints can be suspected > 1.5 kb downstream of *SOX9* and in a 24 bp region 890 bp upstream of the gene, including the entire coding region of *SOX9* and the promoter (compared with the highly homologous sequence of *SOX9* promoters in humans and mouse [11]). This information suggests that the extra copy of the *SOX9* gene is functionally active, although long-range PCR analyses did not reveal the exact location or orientation of the extra copy of *SOX9*.

## Discussion

Here, we report on a specimen of *C* *. capreolus* with a distinct intersex phenotype. It had cranial outgrowths typical of a buck, and a rear phenotype characteristic of a doe. Close inspection revealed no vaginal opening, abdominal testes and a retro-posed pizzle. Our results of molecular genetic testing demonstrate a case of *SRY*-negative XX sex reversal in the investigated intersex roe deer. Similar cases of *SRY*-negative XX sex reversal have been reported previously for dogs, goats, horses and pigs [12–15] but rarely in wild mammals, such as roe deer [5]. A number of genes other than *SRY* (*e.g. WT1*) have been reported as underlying causes of mammalian sex reversal in humans and mouse models [10], but the genetic causes have usually remained elusive. Several genes known to be involved in sex determination or *SRY*-negative XX sex reversal (*SOX3*, *SOX10, RSPO1, DMRT1*, *WT1, RSPO1* and *FOXL2*) were inferred not to be linked to the phenotype in the present intersex roe deer by sequencing analyses and CNV analyses except of *SOX9*. For this gene, we identified a 3-fold increased gene dosage in this deer.

*SOX9* is a direct downstream target of *SRY* and plays a critical role in male sexual differentiation [16]. *SOX9* mutations can lead to haplo-insufficiency, being responsible for campomelic dysplasia, a skeletal malformation syndrome often associated with XY sex reversal in humans [17]. In contrast, duplication of *SOX9* has been implicated in XX sex reversal [18–20]; its ectopic expression in mice induces testis formation in XX gonads [21]. Together with this information, our findings indicate that the extra copy of *SOX9* is sufficient to initiate testis differentiation in the absence of *SRY*, inferring the intersex phenotype in the present roe deer. In this case, the link between phenotype and genotype serves as an excellent example of how a deep sequencing-based approach can gain insights into naturally occurring genetic defects in wild animals, which also has broad implications for understanding complex sex disorders.

## Materials and Methods

### Roe deer

A one-year-old European roe deer was bagged in Schmallenberg (Germany; 51’14’’ N, 8’22’’ O; 700 m altitude). This deer has an intersex appearance, including short unbranched antlers and a displaced penis brush appearing from the distance as a *Schürze* (characteristic tuft of hair in females located just above the vaginal entry). Upon closer inspection the deer presented abnormal male external genitalia, namely small inguinal testicles which had not descended into the scrotum. DNA was isolated from muscle and kidney of the intersex deer. Two healthy European roe deer specimens with normal sexual appearance (bagged in other regions of Germany) were used as male and female controls, respectively. For whole-genome sequencing, EDTA-blood of a healthy male European roe deer from Hohenstein-Born (Germany; 50’09’’ N, 8’05’’ O; 400 m altitude) was used. Genomic DNA was extracted from different tissues (blood cells, muscle and kidney) using a standard protocol [22].

All investigated European roe deer specimens were killed by gunshots from a distance. JTE is a licensed hunter in Germany (*Jahres Jagdschein Nr. 86/2002, Bundesrepublik Deutschland*). Based on *Rehwild-Abschusspläne* (shooting schedules for roe deer), he has to bag a certain number of roe deer specimen in a given area (*Revier*). Such specimens were used for this investigation without having been killed specifically for this study.

### Whole genome sequencing

High molecular weight genomic DNA was isolated from roe deer ( *C* *. capreolus* ), and DNA integrity was confirmed. A short-insert (300 bp) genomic DNA library was prepared and paired-end sequenced using the Illumina sequencing platform (Illumina HiSeq) according to manufacturer’s instructions. The sequence data generated were verified, and low quality sequences removed. The size of the genome and the level of heterozygosity within the deer sample used for sequencing were estimated by establishing the frequency of occurrence of each 17 bp k-mer within the genomic sequence data set. Genome size was estimated using a modification of the Lander Waterman algorithm, where the haploid genome length in bp is: G = (N×(L-K+1)-B)/D, where N is the read length sequenced in bp, L is the mean length of sequence reads, K is k-mer length (defined here as 17 bp) and B is the number of k-mers occurring less than four times. Heterozygosity was evaluated throughout the genome assembly by assessing the distribution of the k-mer frequency for the sequence data set (see Figure S1 in File S1). Paired-end sequence data was assembled using SOAPdenovo [23] employing a k-mer value of 43 or 63 bases (see Table S1 in File S1). Assembly quality and completeness of each assembly was assessed based on the minimum length of sequence contigs and scaffolds of > 100 bp which contained 50% and 90% of the sequence data (N50 and N90, respectively). Assembled genome scaffolds have been deposited in public genome resource databases (http://bioinfosecond.vet.unimelb.edu.au/GasserData/Deer/deer.html; EMBL study accession PRJEB4372).

Genomic DNA libraries (300 bp insert size) were sequenced on the Illumina platform generating a total of 918,961,602 paired-end sequences (average read length of 96 bases). The genome size was estimated to be ^~^3.54 Gb, suggesting that sequence data permitted an average of 21-fold coverage of the genome. Genomes were assembled using k-mers of 43 or 63 and scaffolds were used to generate a sequence homology BLAST database (http://gasser-research.vet.unimelb.edu.au/Deer/wblast6.html).

### PCR amplification, fragment analysis and Sanger DNA sequencing

For sexing of the investigated European roe deer with intersexual appearance, the amelogenin (*AMEL*) gene was amplified based on the genomic sequence of *Bos taurus* (assembly Baylor Btau_4.6.1/bosTau7, The University of California Santa Cruz (UCSC) Genome browser database) under standard conditions. Fragment analyses were performed as described previously [24] on a Beckman-Coulter CEQ 8000 capillary sequencer following standard protocols and using the included software. The described primer sequences from Pajares et al. (2007) were optimized under our own conditions.

To investigate potential translocation of *SRY* sequences from Y to X chromosome, this gene was amplified by PCR using primers from Takahashi et al. (1998). As internal quality control, a bovine autosomal microsatellite locus *BOVIRBP* (*bovine interphotoreceptor retinoid-binding protein*) [25] was amplified by PCR with published primers [26]. All male control samples showed the respective amplifiable sequences.

In order to search for variants causing intersexuality in the investigated roe deer, Sanger DNA sequencing was performed for the roe deer genes *SOX3* (EMBL accession HG326982), *SOX9* (EMBL accession HG326974), *SOX10* (EMBL accession HG326977), *RSPO1* (EMBL accession HG326976), *FOXL2* (EMBL accession HG326975), *DMRT1* (EMBL accession HG326980), *FGF9* (EMBL accession HG326981), *WT1* (EMBL accession HG326978)*, AR* (EMBL accession HG326983) and *SRY* (EMBL accession HG326979) as yielded by whole genome sequencing. Heretofore, intron-based exon specific primers were designed with Primer Express software v2.0 (Applied Biosystems) and amplified by PCR. Sequences of the PCR products were determined using the BigDye Terminator v 3.1 Cycle Sequencing Kit (Applied Biosystems) and an ABI 3500 XL automated capillary sequencer (Applied Biosystems). Sequence variations were annotated to the roe deer reference sequence yielded from whole-genome sequencing.

To investigate the localization and organization of the extra copy of the *SOX9* gene, the respective regions were analyzed with several primer combinations by long-range PCR analyses using expand high fidelity PCR system (Roche). All primer sequences used in this study are listed in Table S2 in File S1.

### Quantitative real-time PCR

The quantitative real-time PCR was performed to assess the copy number of the genes *SOX3*, *SOX9*, *SOX10*, *RSPO1*, *FOXL2*, *DMRT1*, *FGF9*, *WT1, AR* and *SRY* in the genomic DNA of the intersex roe deer as well as the male and female controls. Primers specific to these genes were designed using Primer Express software v2.0 (Applied Biosystems). All real-time PCR assays were carried out using the KAPA^TM^ SYBR Fast qPCR Master Mix (peqlab) as described by the manufacturer on a StepOnePlus^TM^ Real-time PCR detection system (Applied Biosystems). Baseline and threshold values were set automatically and threshold cycle (Ct) values were determined using OneStepPlus software (Applied Biosystems). Copy numbers were calculated using the ΔΔ-CT method [27] with normalization to the house-keeping gene encoding *albumin*. A healthy female roe deer with normal sexual appearance was used as standard for the PCR assay. All measurements were run in triplicate in at least two independent analyses. A statistical analysis was performed for quantitative real-time PCR results of the entire *SOX9* gene and regions 5’ and 3’ of this gene. The mean gene dosage ratio for the region spanning 0.9kb 5’ to 1.5kb 3’ from *SOX9* gene from the intersex roe deer relative to the female control roe deer was 1.6513±0.1321 (p<0.0001; unpaired t-test).

## Supporting Information

File S1 **Supporting information**. Figure S1, Distribution of the k-mer frequency for the roe deer genomic DNA sequence data set. Table S1, Detailed information of the de novo deer assembly. Table S2, Primer sequences used in this study.
(DOCX)
