# Supplementary material for: SOX9 Duplication Linked to Intersex in Deer
Source: PLoS One. 2013 Sep 6;8(9):e73734. doi: 10.1371/journal.pone.0073734 (PMC3765313; doi:10.1371/journal.pone.0073734)
Supplement: File S1 — Supporting information. Figure S1, Distribution of the k-mer frequency for the roe deer genomic DNA sequence data set. Table S1, Detailed information of the de novo deer assembly. Table S2, Primer sequences used in this study. (DOCX) [file pone.0073734.s001.docx]

**Supporting Information**

**Supporting Figures**

**Figure S1** Distribution of the *k*-mer frequency for the roe deer genomic DNA sequence data set.

**Supporting Tables**

**Table S1** Detailed information of the *de novo* deer assembly.

| **Assembly** | **Deer_SOAPdenovo_k43.scafSeq** | **Deer_SOAPdenovo_k63.scafSeq** |
| --- | --- | --- |
| Number of scaffolds | 1406571 | 3088511 |
| Total size of scaffolds | 2426270293 | 2785377831 |
| Total scaffold length as percentage of assumed genome size | 69 | 79 |
| Longest scaffold | 131768 | 151776 |
| Shortest scaffold | 100 | 100 |
| Number of scaffolds > 1K nt | 317505 | 314210 |
| Percentage of scaffolds > 1K nt | 23 | 10 |
| Number of scaffolds > 10K nt | 65469 | 71540 |
| Percentage of scaffolds > 10K nt | 5 | 2 |
| Number of scaffolds > 100K nt | 21 | 49 |
| Mean scaffold size | 1725 | 902 |
| Median scaffold size | 202 | 127 |
| N50 scaffold length | 9822 | 10458 |
| N90 scaffold length | 870 | 183 |
| scaffold %A | 28 | 29 |
| scaffold %C | 20 | 21 |
| scaffold %G | 20 | 21 |
| scaffold %T | 28 | 29 |
| scaffold %N | 3 | 2 |

**Table S2** Primer sequences used in this study.

| **Genes** | **Primer designations** | **Primer sequences (5’→3’)** | **PCR product sizes [bp]** |
| --- | --- | --- | --- |
| **Fragment analyses** | | | |
| *AMELX/Y* | Ex5_F | ACACCACCAGCCAAACCTCC | X: 287 |
|  | Ex5_R | CCCGCTTGGTCTTGTCTGTTGC ^1^ | Y: 224 |
| *SRY* | deerRG4_F | ggtcaagcgacccatgaa(C/T)gcNtt ^2^ | 216 |
|  | deerRG7_R | ggtcgatacttatagttcgggta(C/T)tt ^2^ |  |
|  | BOVIRBP_F | tgtatgatcaccttctatgcttc ^2^ | 180 |
|  | BOVIRBP_R | gctttaggtaatcatcagatagc ^2^ |  |
| **Sequence analyses*** | | | |
| *AR* | Ex1-1_F | AGCCTGTTGAACTCTTCTCAGCA | 785 |
|  | Ex1-1_R | TGCCTTACACAACTCCTTGGC |  |
|  | Ex1-2_F | AGCAGCGGGAGAGCGAGGGAG | 490 |
|  | Ex1-2_R | GGGGAAAGTTGTAGTATTCGCGA |  |
|  | Ex1-3_F | ACTGCTGAGTATTCCCCTTTCAAGG | 713 |
|  | Ex1-3_R | GGGGAGAGGACTGACTCTAGGCTT |  |
|  | Ex2_F | GCAGTTTAATCCTGAAGATTCAGACTTC | 323 |
|  | Ex2_R | CATAAAATCCCGAGTCCTGATAGAC |  |
|  | Ex3_F | TTGGGCAGGATATGTCTCACAC | 323 |
|  | Ex3_R | CTGGCCTCCTTAGAAGCATCA |  |
|  | Ex4_F | CTCAAGCAGCTTTTCTAACAGGG | 451 |
|  | Ex4_R | GGAAAGGAACAGATGTGGTGC |  |
|  | Ex5_F | ATATAATCTGTTCTCCTTTTACTCCCCA | 354 |
|  | Ex5_R | CATTGCCATGAACCAGGTCTGT |  |
|  | Ex6_F | ACATCAAGCTCTTCTTGGAGAAACTG | 352 |
|  | Ex6_R | AATATTCAGGAGCTGGCTTCTTCC |  |
|  | Ex7_F | CAAGTTTGTGGTCAGAAAACTTGG | 299 |
|  | Ex7_R | TGGCTCTATCAGGCTACTCTCCC |  |
|  | Ex8_F | CCTGGAGACAATTTAATGCACATGT | 442 |
|  | Ex8_R | ATTCCCCAAGGCACTGCAGAG |  |
| *DMRT1* | Ex1_F | AGAACGTACGCGTCCTGCG | 469 |
|  | Ex1_R | GGGCTTTACTCGAAAAATTCGG |  |
|  | Ex2_F | GATTCTCAGACCTTCTCCCTGGA | 392 |
|  | Ex2_R | GGCTCGGAACCATCCCA |  |
|  | Ex3_F | AGGACGGAGGCCGTGTG | 422 |
|  | Ex3_R | GCATGTGTGCGGTTTCAAATATC |  |
|  | Ex4_F | TTATTCATGGGTGAGGACACTTCTAA | 320 |
|  | Ex4_R | GTGCCAGCAGGTATCCCCT |  |
|  | Ex5_F | TGTTGGAACCTAATTGCATACTGG | 315 |
|  | Ex5_R | AAACCCTGTGTATAACGAAATAAGCC |  |
| *FGF9* | Ex1_F | AGTTGGATTTACCTCGCCGAGT | 418 |
|  | Ex1_R | AGCTGCGGTCCGAGCCTA |  |
|  | Ex2_F | AAGATGGTTCCCTAGTCAGAGCTC | 273 |
|  | Ex2_R | GAAAGAGTGATTCCCAGCAAATG |  |
|  | Ex3_F | GGGTTAAAATCGGTATATTCTCACCA | 408 |
|  | Ex3_R | GGACCCACCGCGTGAA |  |
| *FOXL2* | Ex1-1_F | GTGAACGGACTTGTGCGCC | 523 |
|  | Ex1-1_R | GGTAGTTGCCCTTCTCGAACATG |  |
|  | Ex1-2_F | TCAGCCTCAACGAGTGCTTCAT | 515 |
|  | Ex1-2_R | CAGACCGTTGTACGAGTTCACCA |  |
|  | Ex1-3_F | CTCGTACGGGCCGTACTCG | 428 |
|  | Ex1-3_R | AGAGCTAGGAGGCGCAGAG |  |
| *RSPO1* | Ex2_F | TCTCCTGGGTAGTGCACCAGAT | 579 |
|  | Ex2_R | ATCACAGAATGAAATGGCCGAA |  |
|  | Ex3_F | CACATTCGTTCTGTAGGCTGAAAC | 392 |
|  | Ex3_R | CTCTTGCAGCCCATCAACAGT |  |
|  | Ex4_F | CGGCAGTGGCAGCTCCA | 351 |
|  | Ex4_R | GGCTCTCTCCGCACGGAG |  |
|  | Ex5_F | TATGCCCTCTGGACCATCAGG | 392 |
|  | Ex5_R | GGTAGGAAACAGAGGCGATCAGA |  |
|  | Ex6_F | GAGGAGGCCTGGAGGGTACC | 369 |
|  | Ex6_R | ACAGCGGCACTGGACTCCTT |  |

**Table S2** Continued.

| **Genes** | **Primer designations** | | **Primer sequences (5’→3’)** | | **PCR product sizes [bp]** | |
| --- | --- | --- | --- | --- | --- | --- |
| **Sequence analyses*** | | | | | | |
| *SOX3* | | 5’UTR_F | | GTATATAAGAAGCCCGGCGAGC | | 1470 |
|  | | 3’UTR_R | | GCAACAGTCCCAGGCAAGC | |  |
|  | | Ex1-1_F | | CAGGCAAgAGtAGTGCGAACG | | 512 |
|  | | Ex1-1_R | | TTCACGTGTGTGTACGTGTCCA | |  |
|  | | Ex1-2_F | | CCAAGATGCACAACTCCGAGAT | | 380 |
|  | | Ex1-2_R | | GCTGCTCATGGTCGCGG | |  |
|  | | Ex1-3_F | | GTGAACGGCTGGGCCAA | | 641 |
|  | | 3’UTR_R | | GCAACAGTCCCAGGCAAGC | |  |
| *SOX9* | | Ex1_F | | CGCCTCGAGTGCCCGA | | 574 |
|  | | Ex1_R | | CGCCGCGGAGCCCA | |  |
|  | | Ex2_F | | TTCATCCGCAGGGTTTCCA | | 618 |
|  | | Ex2_R | | TGTCCCGATGCAGAGCCTC | |  |
|  | | Ex3-1_F | | GTTAATCATTCTGTGCCTTATCTCGG | | 753 |
|  | | EX3-1_R | | CTTGATGTGCGTGCGCTG | |  |
|  | | Ex3-2_F | | GTGTGGATGTCCAAGCAGCAG | | 683 |
|  | | Ex3-2_R | | ATGTCCGAAGGGTCGCGT | |  |
| *SOX10* | Ex2-1_F | | CTTAGGGCTGTGCCTGAGTCG | | 456 | |
|  | Ex2-1_R | | CCGTTGACGCGCACCG | |  | |
|  | Ex2-2_F | | ATGACAAGTTCCCCGTGTGCA | | 296 | |
|  | Ex2-2_R | | ATTCCACCCGCGGGTAGAG | |  | |
|  | Ex3_F | | CTAGACCAGACAGCGCGACTCC | | 471 | |
|  | Ex3_R | | TCTCAAGTGCTGCTCCAGCCTT | |  | |
|  | Ex4_F | | CGTCTGAAAGTGAACCATCAGCCACT | | 614 | |
|  | Ex4_R | | GTAGTGGGGCAGACTGAGGGAGGTGTA | |  | |
| *SRY* | Ex1-1_F | | GGTAAGAACAACTTATTTAACAGCACGAT | | 519 | |
|  | Ex1-1_R | | TTGAAGAGTCTGCAGGAAGCAAT | |  | |
|  | Ex1-2_F | | GGTATGAGTGGAAAAGGCTTACAGAT | | 489 | |
|  | Ex1-2_R | | AGTCACAGGTGAAATTGTAGTAAAATTGAGA | |  | |
| *WT1* | Ex1_F | | GGCATCTGGGCCAAGTTAGG | | 649 | |
|  | Ex1_R | | ACCGGTTAAGAACTGTGGTCAGG | |  | |
|  | Ex2_F | | ACTGGACCCGGCTTCCCT | | 335 | |
|  | Ex2_R | | GCTGTGGGTTGGAAATTCCTG | |  | |
|  | Ex3_F | | TTGCGCCCTGCACAGC | | 262 | |
|  | Ex3_R | | CCGCCAGCTCGAGCATC | |  | |
|  | Ex4_F | | CATGTGGAGATCAGTGGGATGA | | 360 | |
|  | Ex4_R | | CACGCCTATTCTACAACTGTGCTCT | |  | |
|  | Ex5_F | | TCAGGGACCCAGTTCAGCA | | 287 | |
|  | Ex5_R | | GATTACCCGTCATCAGTCCTAACTC | |  | |
|  | Ex6_F | | CCCGGGAACTCTGAGAGGA | | 307 | |
|  | Ex6_R | | TGACACAGGAGCCAATGGTTAG | |  | |
|  | Ex7_F | | TGGTTTCAGGCATCGCATC | | 355 | |
|  | Ex7_R | | TGGCAGTGACAGCTAGAAGCAG | |  | |
|  | Ex8_F | | GTCCTTTCAGTGCCAGTAAGAGAAGT | | 342 | |
|  | Ex8_R | | TTTTAAAGAGAAGGTAAATCCCTAGCAA | |  | |
|  | Ex9_F | | AGGTATGGCTGGAAATGCCC | | 285 | |
|  | Ex9_R | | ATCTCTCACATCACAAGCAAATCC | |  | |
|  | Ex10_F | | AGGTGCTGCCATATCAAGGC | | 391 | |
|  | Ex10_R | | GACAGGCGAGTGAGGAGGAAT | |  | |
| **Real time PCR analyses** | | | | | | |
| *AR* | Ex2RT_F | | TTTGGAGACCACCAGGGACCA | | 152 | |
|  | Ex2RT_R | | CTTCAGCGGCTCTTTTAAAAAAGAC | |  | |
| *DMRT1* | Ex3RT_F | | GTCATCCAGGATATTCCTGCTGTC | | 148 | |
|  | Ex3RT_R | | TGGAGTACTGCGGGTAGTTGTAGAG | |  | |
|  | Ex4RT_F | | AACCCGGAGAACCTCCACG | | 139 | |
|  | Ex4RT_R | | CGCTCGTTTTGGCTTCCG | |  | |
| *FGF9* | Ex1RT_F | | GGGTCAGTCCGAAGCAGGAG | | 142 | |
|  | Ex1RT_R | | TCCCTGGATAGTACCGTTGGG | |  | |
|  | Ex2RT_F | | CAGGGATCCTGGAGTTCATCAGTAT | | 169 | |
|  | Ex2RT_R | | GAAAGAGTGATTCCCAGCAAATG | |  | |

**Table S2** Continued.

| **Genes** | | **Primer designations** | **Primer sequences (5’→3’)** | **PCR product sizes [bp]** |
| --- | --- | --- | --- | --- |
| **Real time PCR analyses** | | | | |
| *FOXL2* | Ex1RT_F | | TCAGCCTCAACGAGTGCTTCAT | 148 |
|  | Ex1RT_R | | GGTAGTTGCCCTTCTCGAACATG |  |
| *RSPO1* | Ex4RT_F | | GATGCAAGATTGAGCACTGCGA | 148 |
|  | Ex4RT_R | | GGGCTGCCGCACTCCAT |  |
| *SOX3* | Ex1RT_F | | GTGAACGGCTGGGCCAA | 112 |
|  | Ex1RT_R | | GCTGCTCATGGTCGCGG |  |
| *SOX9* | 800kb 5‘RT_F | | AAGGCAGTGTTCCAAACTGCTAG | 121 |
|  | 800kb 5‘RT_R | | TCTAAGGATCACAGTAGGAATCTCTCGT |  |
|  | 1kb 5‘RT_F | | CTTTCATTGATTCTCTGCGCC | 107 |
|  | 1kb 5‘RT_R | | CCGTTCCTCTGTAATAATCCGC |  |
|  | 0.9kb 5‘RT_F | | TGCTTCAAAAGACAATTTCTAAGCAC | 124 |
|  | 0.9kb 5‘RT_R | | GAATACTGCGAAGCCAGTTAAGTCC |  |
|  | 0.36kb 5’UTR RT_F | | GGAGCTTGAAACTGACCTGGAAC | 111 |
|  | 0.36kb 5’UTR RT_R | | CAATCGCCCCCGCTTTC |  |
|  | | Ex1RT_F | ATGAATCTCCTGGACCCCTTCATGAA | 128 |
|  | | Ex1RT_R | GTGTCGGAGCCGGAGCCCGAAGGGCA |  |
|  | | IVS1 RT_F | ggttcggcagaagcagcttt | 104 |
|  | | IVS1 RT_R | GGCCTTTGCTACTGGATATTTACCC |  |
|  | | Ex2RT_F | GCGTGCAGCACAAGAAGGAT | 142 |
|  | | Ex2RT_R | GCCTGCAGCGCCTTGAA |  |
|  | | IVS2 RT_F | gttaatcattctgtgccttatctcgg | 114 |
|  | | IVS2 RT_R | CTGCGCAGAGGCTCTGGTC |  |
|  | | Ex3RT_F | CTACATGAGCCCGGCGCAGCGGCCC | 116 |
|  | | Ex3RT_R | GCTGTGTGTACACCGGCTGTTCCCA |  |
|  | | 1kb 3’UTR RT_F | AGTGCCCCAGCACACTGC | 115 |
|  | | 1kb 3’UTR RT_R | GAAGGTTAACTGCTGGTGTTCAGAG |  |
|  | | 1.5kb 3‘RT_F | ATGTAGTGTATCACTGAGTCATTCGCA | 143 |
|  | | 1.5kb 3‘RT_R | TCCCCTCTCTCTCCAAGAGACA |  |
|  | | 10kb 3‘RT_F | CTGACTCAACACAGCATCGGA | 134 |
|  | | 10kb 3‘RT_R | TCCACACTCTGTGCCCAGG |  |
| *SOX10* | | Ex2RT_F | ATGGCAGAGGAGCAGGACCT | 189 |
|  | | Ex2RT_R | GCCGTCCTGCTGTTCCTTCT |  |
|  | | Ex4RT_F | TCCGACCACCCCAAAGACA | 144 |
|  | | Ex4RT_R | ATGTTGGACATTACCTCATGGCTG |  |
| *SRY* | | Ex1RT_F | GGTATGAGTGGAAAAGGCTTACAGAT | 159 |
|  | | Ex1RT_R | TTGAAGAGTCTGCAGGAAGCAAT |  |
| *WT1* | | Ex1RT_F | GTCATCCAGGATATTCCTGCTGTC | 148 |
|  | | Ex1RT_R | TGGAGTACTGCGGGTAGTTGTAGAG |  |
|  | | Ex7RT_F | AACCCGGAGAACCTCCACG | 139 |
|  | | Ex7RT_R | CGCTCGTTTTGGCTTCCG |  |
| *albumin* | | Ex12RT_F | AGTCACCAAATGCTGCACGG | 141 |
|  | | Ex12RT_R | GCATCGGGAAGGGTGCATAT |  |
| **Long-range PCR analyses** | | | | |
| *SOX9* | | Ex3RT_F | CTACATGAGCCCGGCGCAGCGGCCC | ≥1900 |
|  | | 5‘-768_R | GGACTTAACTGGCTTCGCAGTATTC |  |
|  | | Ex3RT_F | CTACATGAGCCCGGCGCAGCGGCCC | ≥2000 |
|  | | 3’UTR+1504_F | ATGTAGTGTATCACTGAGTCATTCGCA |  |
|  | | 5‘-998_F | CTTTCATTGATTCTCTGCGCC | 255^#^ |
|  | | 5‘-768_R | GGACTTAACTGGCTTCGCAGTATTC |  |
|  | | Ex3RT_F | CTACATGAGCCCGGCGCAGCGGCCC | 1776 |
|  | | 3‘UTR+1625_R | TCCCCTCTCTCTCCAAGAGACA |  |

^1^ Pajares et al. 2007

^2^ Takahashi et al. 1998

* Primers used for sequence analyses are tailed with M13 Tails (F: GTAAAACGACGGCCAGT; R: CAGGAAACAGCTATGAC)

^#^ control systems for long range PCR
